# Expression of nitric oxide synthase isoforms in the dorsal horn of monoarthritic rats: effects of competitive and uncompetitive *N*-methyl-D-aspartate antagonists

**DOI:** 10.1186/ar2208

**Published:** 2007-05-23

**Authors:** Claudio Infante, Marcelo Díaz, Alejandro Hernández, Luis Constandil, Teresa Pelissier

**Affiliations:** 1 Program of Physiopathology, Institute of Biomedical Sciences (ICBM), Faculty of Medicine, University of Chile, Ave. Salvador 486, P.O. Box 16038 Santiago 9, Santiago, Chile; 2 Laboratory of Neurobiology, Department of Biology, Faculty of Chemistry and Biology, University of Santiago of Chile (USACH), Ave. B. Libertador B. O'Higgins 3363, P.O. Box 40 Correo 33, Santiago, Chile; 3 Program of Molecular and Clinical Pharmacology, Institute of Biomedical Sciences (ICBM), Faculty of Medicine, University of Chile, Independencia 1027, P.O. Box 70000 Santiago 7, Santiago, Chile

## Abstract

Chronic pain is associated with *N*-methyl-D-aspartate (NMDA) receptor activation and downstream production of nitric oxide, which has a pivotal role in multisynaptic local circuit nociceptive processing in the spinal cord. The formation of nitric oxide is catalyzed by three major nitric oxide synthase (NOS) isoforms (neuronal, nNOS; inducible, iNOS; endothelial, eNOS), which are increased in the spinal cord of rodents subjected to some tonic and chronic forms of experimental pain. Despite the important role of NOS in spinal cord nociceptive transmission, there have been no studies exploring the effect of NMDA receptor blockade on NOS expression in the dorsal horn during chronic pain. Furthermore, NOS isoforms have not been fully characterized in the dorsal horn of animals subjected to arthritic pain. The aim of this work was therefore to study the expression of nNOS, iNOS and eNOS in the dorsal horns of monoarthritic rats, and the modifications in NOS expression induced by pharmacological blockade of spinal cord NMDA receptors. Monoarthritis was produced by intra-articular injection of complete Freund's adjuvant into the right tibio-tarsal joint. At week 4, monoarthritic rats were given either the competitive NMDA antagonist (±)-3-(2-carboxypiperazin-4-yl)-propyl-1-phosphonic acid (CPP) or the uncompetitive NMDA antagonist ketamine. After 6 and 24 hours, animals were killed and posterior quadrants of the lumbar spinal cord were dissected. Sample tissues were homogenized and subjected to immunoblotting with anti-nNOS, anti-iNOS or anti-eNOS monoclonal antibodies. The nNOS isoform, but not the iNOS and eNOS isoforms, were detected in the dorsal horns of control rats. Monoarthritis increased the expression of nNOS, iNOS and eNOS in the dorsal horns ipsilateral and contralateral to the inflamed hindpaw. Intrathecal administration of CPP and ketamine reduced nNOS expression in monoarthritic rats but increased the expression of iNOS and eNOS. Results suggest that blockade of spinal cord NMDA receptors produces complex regulatory changes in the expression of NOS isoforms in monoarthritic rats that may be relevant for nitridergic neuronal/glial mechanisms involved in the pathophysiology of monoarthritis and in the pharmacological response to drugs interacting with NMDA receptors.

## Introduction

Hyperalgesia, one of the main features of chronic pain, develops closely associated with increased glutamatergic neurotransmission in the dorsal horn of the spinal cord, especially to *N*-methyl-D-aspartate (NMDA) receptor activation. Accordingly, a variety of NMDA receptor antagonists, acting on different sites of the receptor, have demonstrated antinociceptive efficacy on chronic experimental inflammatory and neuropathic pain syndromes [1-5]. NMDA receptor activation is followed by downstream modifications of intracellular signaling, including activation of nitric oxide synthase (NOS), which catalyzes the formation of nitric oxide from arginine. Nitric oxide is a gaseous mediator that seems to have a pivotal role in multisynaptic local circuit nociceptive processing in the spinal cord. It is generated by three major NOS isoforms: nNOS (neuronal NOS) and eNOS (endothelial NOS), which are calcium-dependent constitutive enzymes, and iNOS (inducible NOS), which a calcium-independent inducible isoform [6-8].

Intrathecally administered NMDA induces short-term hyperalgesia, whereas systemic and intrathecal administration of the non-selective NOS inhibitor *N*^ω^-nitro-L-arginine methyl ester (L-NAME) blocks NMDA-induced hyperalgesia, suggesting that the generation of nitric oxide contributes to this response [9]. In addition, intrathecal L-NAME prevents thermal pain hypersensitivity in rats after carrageenan injection [10] and sciatic nerve constriction-induced injury [11], as well as thermal and mechanical hypersensitivity induced in mice by the intraplantar administration of complete Freund's adjuvant (CFA) [12]. Besides, increased expression of one or more of the three NOS isoforms has been shown in the spinal cord of rodents after carrageenan injection into a hindpaw [13], intraplantar injection of CFA [12] and formalin [14], and intradermal injection of capsaicin [15]. However, in these models of tonic experimental pain, only fast and short-term hyperalgesia and allodynia are tested. With regard to changes in NOS expression in long-term experimental models of chronic pain, the available data refer only to the spinal nerve ligation model in rats [16,17], whereas expression of NOS in the spinal cord in rat models of arthritic pain was only partly studied [18]. It has been shown that monoarthritic pain is highly sensitive to NMDA antagonists [19] and to L-NAME [20], suggesting an involvement of the nitric oxide/cyclic GMP cascade in downstream NOS activation in the spinal cord. However, there have been no studies exploring the effect of NMDA receptor blockade on NOS expression in the dorsal horn. The aim of this work was therefore to study the expression of nNOS, iNOS and eNOS in the dorsal horns of monoarthritic rats, and to explore how the expression of NOS isoforms in this model of chronic pain is modified by pharmacological blockade of spinal cord NMDA receptors with competitive and uncompetitive antagonists.

## Materials and methods

### Animals

Investigations were performed on 26 young adult male Sprague-Dawley rats weighing 300 to 350 g. The animals were housed in a room with a 12-hour light/dark cycle with food and water *ad libitum*. All experimental protocols and animal management were in accordance with the Ethical Guidelines for Investigations of Experimental Pain in Conscious Animals [21] and were approved by the Committee for the Ethical Use of Experimental Animals, Faculty of Medicine, University of Chile.

### Monoarthritis

Monoarthritis was induced by intra-articular injection (50 μl) of CFA (60 mg of killed *Mycobacterium butyricum *suspended in a mixture of 6 ml of paraffin oil, 4 ml of 0.9% NaCl and 1 ml of Tween 80) into the right tibio-tarsal joint, as described by Butler and colleagues [22]. Control rats were injected intra-articularly with the vehicle used to suspend mycobacteria. Monoarthritis and control rats were used 4 weeks after the administration of adjuvant or vehicle.

### Protocols

The animals were divided into four groups receiving intrathecally, by the percutaneous route [23], 10 μl of saline solution or drug, as follows: (1) control group (*n *= 4), normal rats receiving saline; (2) monoarthritic/saline group (*n *= 6), monoarthritic rats receiving saline; (3) monoarthritic/CPP group (*n *= 8), monoarthritic rats receiving 100 μg of the competitive NMDA receptor antagonist (±)-3-(2-carboxypiperazin-4-yl)-propyl-1-phosphonic acid (CPP); (4) monoarthritic/ketamine group (*n *= 8), monoarthritic rats receiving 100 μg of the uncompetitive NMDA receptor antagonist ketamine. Drugs and saline were administered three times every 2 hours.

At 6 and 24 hours after the first administration of drug or saline, animals were deeply anesthetized with 60 mg/kg sodium pentobarbital and killed by decapitation. The lumbar spinal cord was then removed (L2–L4 vertebrae level), and the right and left posterior quadrants containing the I–VI laminae were dissected. Spinal cord dissection was made under a × 5 magnifying glass in a Petri dish containing saline solution at 4°C. Each spinal segment was kept in an Eppendorf tube and stored at -20°C.

### Immunoblotting

**Figure 1:**
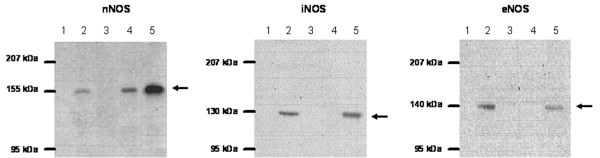
Western blotting of NOS isoforms in lumbar dorsal horn of normal and monoarthritic rats. Left panel, neuronal nitric oxide synthase (nNOS); middle panel, inducible nitric oxide synthase (iNOS); right panel, endothelial nitric oxide synthase (eNOS). Lane 4, lumbar dorsal horn of normal rat; lane 5, lumbar dorsal horn of monoarthritic rat. The positive and the negative controls are shown in lanes 2 and 3, respectively (for a description of the positive and negative controls see the Materials and methods section). The standards for molecular mass were run in lane 1, as shown by the adjacent marks. Arrows indicate the position of bands detected by the monoclonal antibodies at the expected sizes for nNOS (155 kDa), eNOS (140 kDa) and iNOS (130 kDa).

A first screening for the presence of the three NOS isoforms in the dorsal horn of the spinal cord of normal and monoarthritic rats was performed by Western blot assay, which permitted the determination of the size and the fractionation degree of each NOS isoform as well as the sensitivity and specificity of the monoclonal antibodies used (Figure 1). To evaluate the specificity of antibodies, the positive and negative controls provided by the manufacturer were used. The positive controls for nNOS, iNOS and eNOS were, respectively, rat pituitary homogenate, macrophages cultured in a medium with interferon-γ and lipopolysaccharide, and human vascular endothelium (Transduction Laboratories, Lexington, KY, USA). The positive control for anti-eNOS antibody was used as a negative control for both nNOS and iNOS, and the positive control for anti-iNOS antibody was used as a negative control for eNOS. Sample tissues were homogenized in four volumes of cold Tris-HCl buffer (50 mM Tris-HCl, 0.1 mM EDTA, 12 mM 2-mercaptoethanol, 2 μM leupeptin, 1 μM pepstatin and 1 μM phenylmethylsulfonyl fluoride) and centrifuged at 3,000*g *for 20 minutes at 4°C. In the Western blot assay, 5 μg of total protein from each sample, determined by the method of Lowry [24], were separated by electrophoresis in 8% polyacrylamide gels (SDS-PAGE) and thereafter transferred to poly(vinylidene difluoride) membranes (Immobilon^®^; Millipore, Marlborough, MA, USA) by means of electroblotting with Mini Protean Cell III equipment (Bio-Rad Laboratories, Richmond, CA, USA). Afterwards the membranes were incubated at 4°C in blocking solution made of 6% dried non-fat milk in TBS-T buffer (20 mM Tris-HCl, 137 mM NaCl and 0.05% Tween 20, pH 7.6). The membranes were then incubated for 2 hours with either anti-nNOS, anti-iNOS or anti-eNOS monoclonal antibody, diluted 1:4,000, 1:4,000 and 1:1,000 respectively, dissolved in 6% dried non-fat milk in TBS-T buffer. After being rinsed with TBS-T buffer, the membranes were incubated overnight with horseradish peroxidase-conjugated secondary antibody (Pierce Biotechnology Inc., Rockford, IL, USA), diluted 1:10,000 in 6% dried non-fat milk in TBS-T buffer. A positive reaction was identified with enhanced chemiluminescence (SuperSignal West Pico Chemiluminescent Substrate; Pierce Biotechnology Inc.). Membranes were exposed to X-ray film (CL-Xposure film; Pierce Biotechnology Inc.) and the films were then scanned to determine the optical density of each band with the program Uni-Scan (Lab Systems, Espoo, Finland). The specificity of the second antibody was also evaluated by incubating the tissue samples with the second antibody alone, thus permitting the detection of possible unspecific interactions between the second antibody with some proteins of the sample.

Because of the high specificity of the antibodies employed and the absence of unspecific interactions of the second antibody with tissue samples (Figure 1), it was possible to perform all determinations of NOS isoforms by means of an immunodot blot. Proteins (5 μg) from the supernatant homogenates of the right and left dorsal horns were added directly to poly(vinylidene difluoride) membranes by means of a dot-blot device (Bio-Rad Laboratories). Thereafter, the membranes were incubated with primary and secondary antibodies, with the same procedures as those described above for Western blotting. Membranes were exposed to X-ray film and the films were scanned to determine the optical density of each dot. Densitometric measurements in every experiment were standardized by dividing the results for a unique internal control for each isoform of NOS, which were systematically employed in every experiment.

### Statistical analysis

All data are presented as means ± SEM. The statistical significance of differences between groups was determined with the Kruskal–Wallis test (InStat 3.00; GraphPad Software Inc., San Diego, CA, USA). Differences were considered significant at *p *< 0.05.

## Results

### NOS expression in control and monoarthritic rats

**Figure 2:**
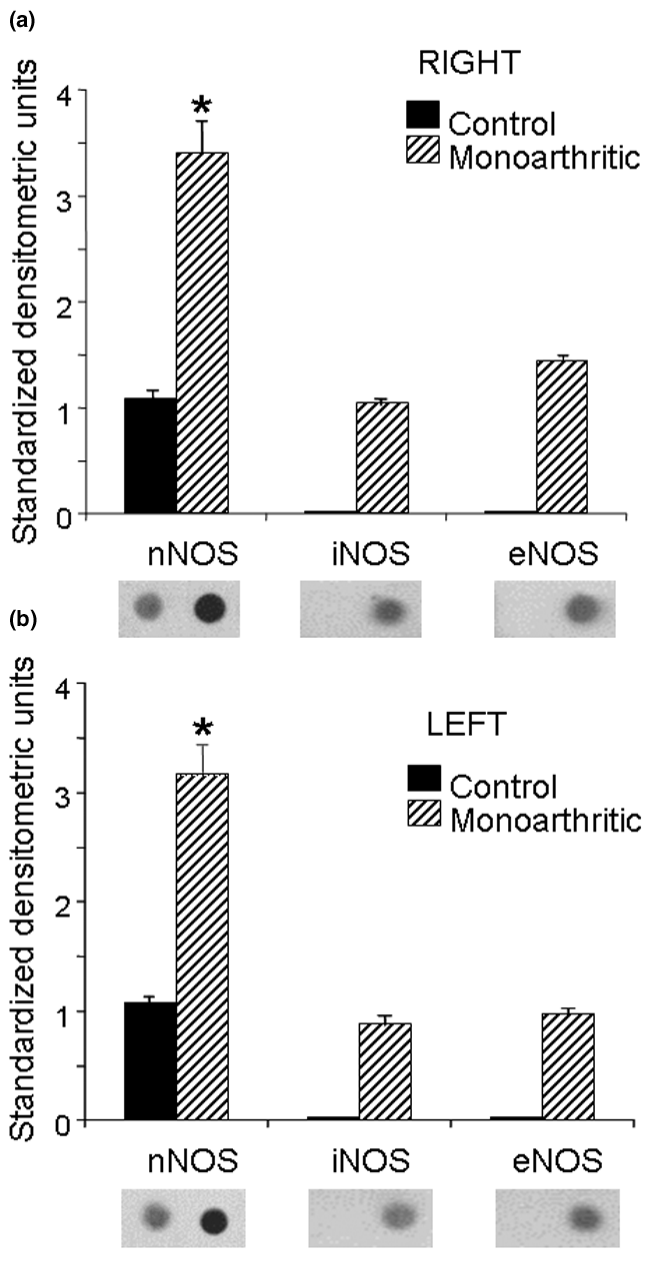
Expression of nitric oxide synthase (NOS) isoforms in dorsal horns of control and monoarthritic rats. **(a) **Right dorsal horn; **(b) **left dorsal horn. Monoarthritis was induced by intra-articular injection of complete Freund's adjuvant (CFA) into the right tibio-tarsal joint; control rats received the vehicle of CFA. The expression of NOS isoforms was determined 4 weeks later. Bar graphs show the expression of the neuronal (nNOS), inducible (iNOS) and endothelial (eNOS) isoforms in standardized units (means ± SEM); below each bar graph a representative example of immunodot blotting is shown. Only nNOS was expressed in control rats. In monoarthritic rats the expression of the three NOS isoforms was found to be increased bilaterally (*p < 0.05 compared with control; Kruskal–Wallis test).

The nNOS isoform was detected bilaterally in dorsal horns of control rats. In monoarthritic rats nNOS expression was increased in the dorsal horns ipsilateral (right) and contralateral (left) to the inflamed hindpaw, in comparison with those of control rats. In the ipsilateral dorsal horn nNOS expression increased by 216% (Figure 2a), and in the contralateral dorsal horn the increase in nNOS expression was 194%, the difference between the sides being not statistically significant (Figure 2b). Then, despite the unilateral condition of the arthritic disease induced in the present experiments, increments in nNOS expression were similar in the ipsilateral and contralateral dorsal horns (Figure 2a, b).

Figure 2 also shows that the iNOS and eNOS isoforms could not be detected in dorsal horns from normal control rats. However, these two NOS isoforms were clearly expressed in the spinal cord dorsal horn of monoarthritic rats, presenting a bilateral expression pattern. The expression of iNOS and eNOS in dorsal horns of monoarthritic rats was less than that of nNOS (Figure 2a, b).

### Effect of NMDA antagonists in NOS expression in monoarthritic rats

**Figure 3:**
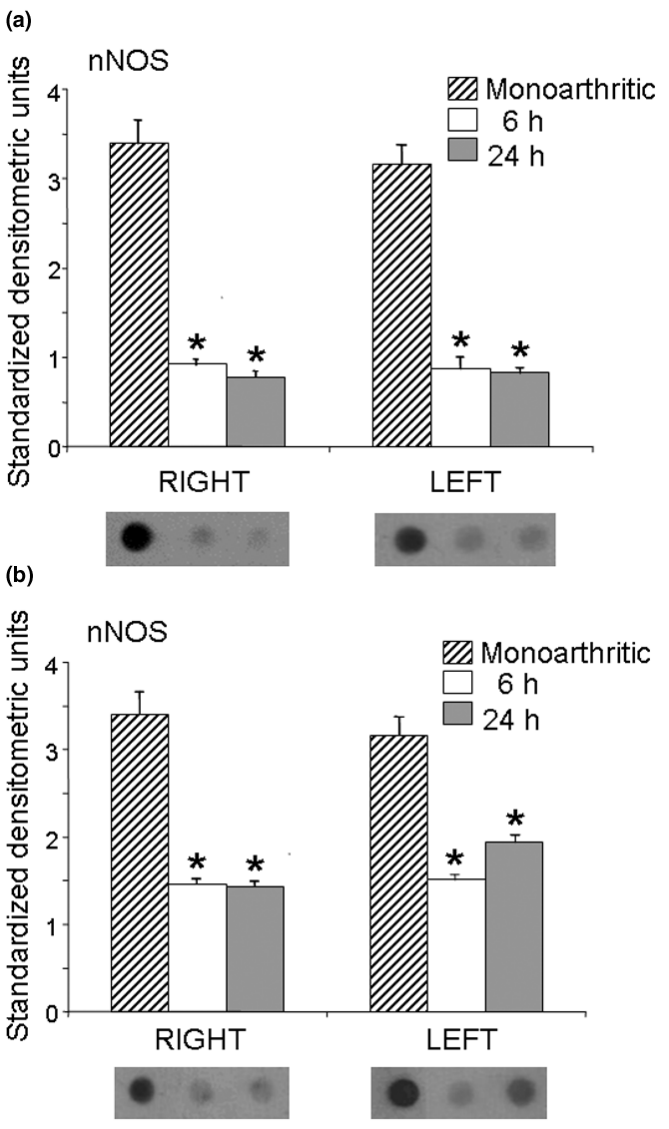
Expression of nNOS in the right and left dorsal horns of monoarthritic rats. Neuronal nitric oxide synthase (nNOS) expression was measured 6 and 24 hours after intrathecal administration of (±)-3-(2-carboxypiperazin-4-yl)-propyl-1-phosphonic acid (CPP) **(a) **and ketamine **(b)**. Bar graphs show nNOS expression in standardized units (means ± SEM); below each bar graph a representative example of immunodot blotting is shown. Both CPP and ketamine decreased nNOS expression compared with saline-injected monoarthritic controls (*P < 0.05 compared with control; Kruskal–Wallis test).

Intrathecal administration of CPP and ketamine decreased nNOS expression in monoarthritic rats. In the ipsilateral (right) dorsal horn nNOS expression decreased by 73% and 77% at 6 and 24 hours after the administration of CPP, respectively, whereas in the contralateral (left) dorsal horn nNOS decreased by 73% and 75% during the same periods (Figure 3a). In the right dorsal horn nNOS decreased by 57% and 59% at 6 and 24 hours after intrathecal administration of ketamine, respectively, whereas in the left dorsal horn nNOS decreased by 53% and 39% during the same periods (Figure 3b). Intrathecal administration of saline produced no significant effects.

**Figure 4:**
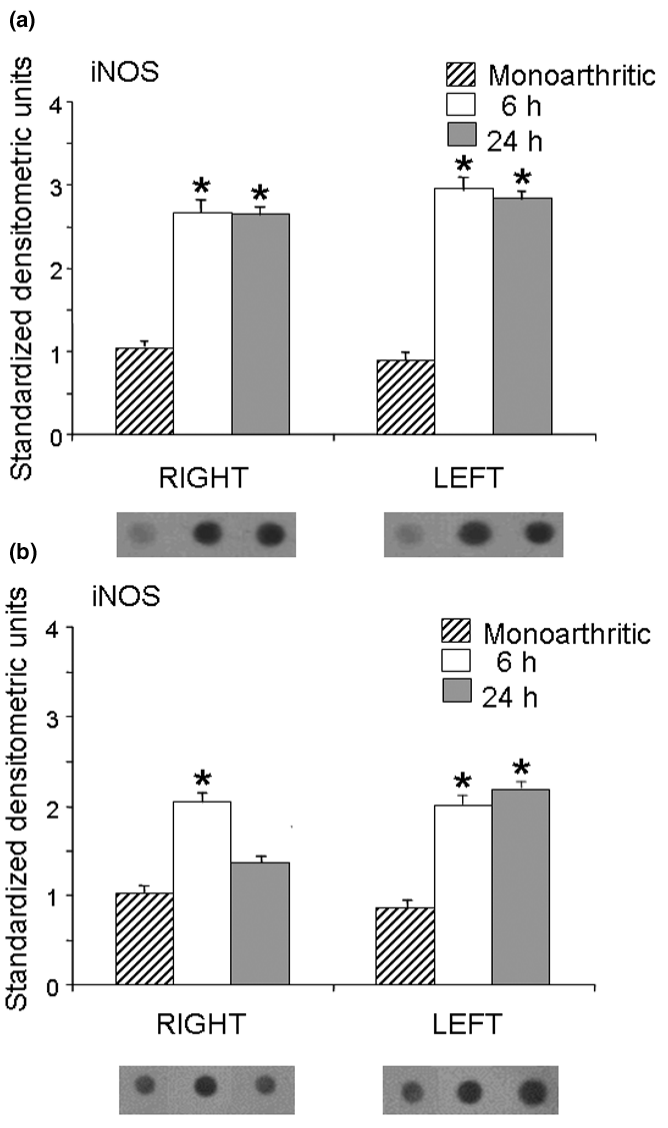
Expression of iNOS in the right and left dorsal horns of monoarthritic rats. Inducible nitric oxide synthase (iNOS) expression was measured 6 and 24 hours after intrathecal administration of (±)-3-(2-carboxypiperazin-4-yl)-propyl-1-phosphonic acid (CPP) **(a) **and ketamine **(b)**. Bar graphs show iNOS expression in standardized units (means ± SEM); below each bar graph a representative example of immunodot blotting is shown. Both CPP and ketamine increased iNOS expression compared with saline-injected monoarthritic controls (*P < 0.05 compared with control; Kruskal–Wallis test).

Intrathecal administration of CPP and ketamine increased the expression of the inducible iNOS isoform in monoarthritic rats, in comparison with monoarthritic controls receiving intrathecal saline. In the ipsilateral (right) dorsal horn iNOS expression increased by 155% and 154% at 6 and 24 hours after the administration of CPP, respectively, whereas in the contralateral (left) dorsal horn iNOS increased by 244% and 231% during the same periods (Figure 4a). A similar increasing effect in iNOS expression was also observed after the administration of ketamine; these increases were 102% and 31% in the ipsilateral dorsal horn and 146% and 170% in the contralateral dorsal horn at 6 and 24 hours, respectively, after drug administration (Figure 4b).

**Figure 5:**
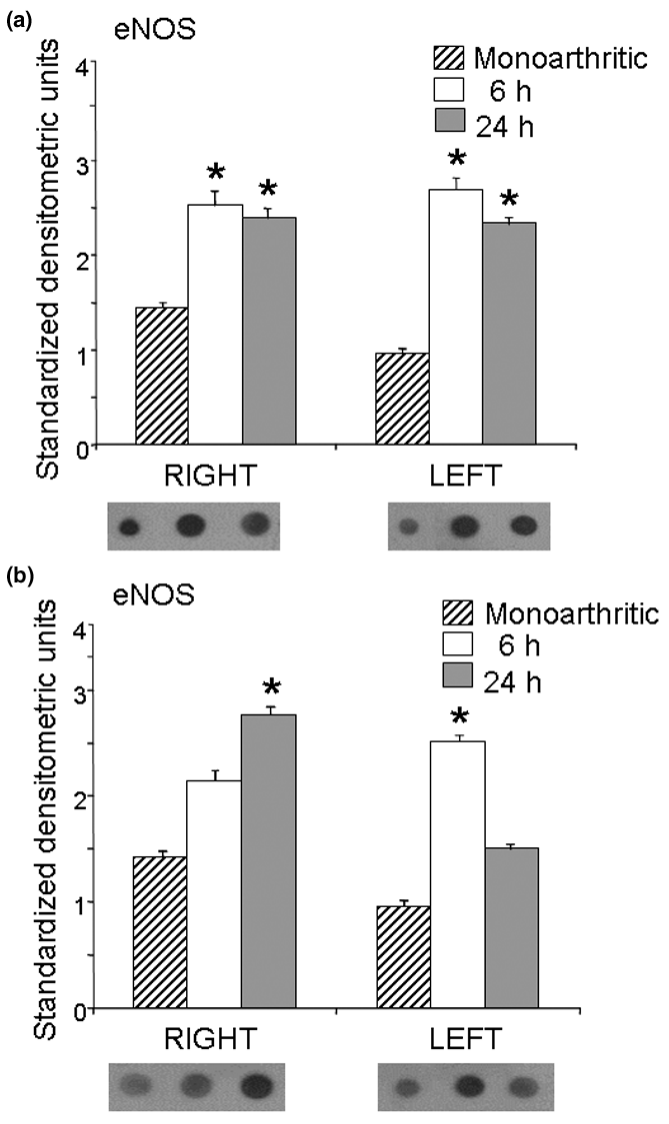
Expression of eNOS in the right and left dorsal horns of monoarthritic rats. Endothelial nitric oxide synthase (eNOS) expression was measured 6 and 24 hours after intrathecal administration of (±)-3-(2-carboxypiperazin-4-yl)-propyl-1-phosphonic acid (CPP) **(a) **and ketamine **(b)**. Bar graphs show eNOS expression in standardized units (means ± SEM); below each bar graph a representative example of immunodot blotting is shown. Both CPP and ketamine increased eNOS expression compared with saline-injected monoarthritic controls (*P < 0.05 compared with control; Kruskal–Wallis test).

Intrathecal administration of CPP and ketamine also increased the expression of the constitutive eNOS isoform in monoarthritic rats in comparison with monoarthritic controls receiving intrathecal saline. Thus, intrathecal CPP increased ipsilateral (right dorsal horn) eNOS expression by 74% and 66% at 6 and 24 hours, respectively, after drug administration, whereas contralateral (left dorsal horn) eNOS expression increased by 183% and 144% during the same periods (Figure 5a). With regard to ketamine, in the ipsilateral dorsal horn eNOS increased by 48 and 93% at 6 and 24 hours, respectively, after intrathecal administration of ketamine, whereas in the contralateral dorsal horn eNOS increased by 161% and 56% during the same periods (Figure 5b).

## Discussion

Results showed that the nNOS isoform was expressed in the lumbar dorsal horn of intact control rats, whereas the iNOS and eNOS isoforms could not be detected in the same spinal cord regions of these animals. This is in accordance with earlier studies showing moderate expression of nNOS in the spinal cord of intact rats [25]. In addition, the present results showed that the three major NOS isoforms were expressed bilaterally in the dorsal horns of monoarthritic rats, which is in agreement with previous observations that injection of incomplete Freund's adjuvant into the knee joint cavity increased the expression of the nNOS and iNOS isoforms in the lumbar enlargement of rats [18]. Increased expression of nNOS and/or iNOS has also been shown in the lumbar spinal cord of rats after carrageenan injection into a hindpaw [12], intraplantar administration of formalin [14,26] or CFA [12], and intradermal injection of capsaicin [15]; large increases in the expression of nNOS and iNOS have been observed in dorsal horns taken from L4-L6 spinal cord segments of rats subjected to ligation of the sciatic nerve [16,17].

The differences in NOS expression levels found in these studies could be the result of the different procedures used to induce persistent pain (namely long-lasting chronic pain after sciatic nerve ligation and intra-articular administration of Freund's adjuvant, versus tonic pain after administration of carrageenan, formalin or capsaicin), the different time course of the pain induced, and/or the different times within the hyperalgesic process at which NOS determinations were made, but they could also be the result of differential glial activation in the spinal cord. In fact, a role of spinal glia has recently been described for the initiation and early maintenance of inflammatory pain facilitation in monoarthritic rats [27]. The fact that levels of eNOS in the spine have been shown to increase in astrocytes but not in neurons after the injection of carrageenan [28] suggests the participation of spinal cord glia in the increases in eNOS reported here. In this regard, the regulation of only the genes encoding iNOS in glia has been well described [29], but more recently reverse transcriptase-mediated polymerase chain reaction and Western blot analyses have revealed that mRNAs encoding the constitutive nNOS and eNOS as well as the corresponding proteins were expressed in human astrocytes [30], thus opening the possibility that glial activation could account, at least in part, for the observed increases in expression of the three NOS isoforms in the dorsal horn of monoarthritic rats. Further investigation will be required to determine to what extent increases in NOS isoforms in dorsal lumbar spinal cord are dependent on neuronal and/or glial function.

Although in the present experiments the inflammation was induced on the right hind limb only, the changes in expression of NOS isoforms were found in the dorsal lumbar horn of both sides, suggesting a functional role for the contralateral innervation, at least during conditions of arthritic pain. In this respect it has been recognized that neurons in the spinal cord receive inputs from the contralateral side that, under normal conditions, are ineffective in generating an active response. However, there exist studies showing that on iontophoretic administration of NMDA or strychnine on one dorsal horn, the neurons of the opposite dorsal horn increased their excitability, thereby indicating that the contralateral input participates in the circuit dynamics of spinal nociceptive transmission [31]. This makes possible a functional role for such crossed connections in neuronal sensitization after unilateral peripheral injury. Support for this hypothesis can be found in the study by Ondarza and colleagues [32] showing a bilateral massive increase in calcitonin gene-related peptide staining (a marker for nociceptive endings) colocalized with GAP-43 (a marker for neurite sprouting) in the dorsal horn of rats subjected to unilateral spinal cord injury, indicating that mechanisms participating in the reorganization of nociceptive neuronal connections in dorsal horn circuits may be bilaterally activated in spite of the unilateral condition of the injury. An alternative explanation for the bilateral expression of NOS isoforms in monoarthritic rats could be that the process of disease spreads to the contralateral side, thereby stimulating the upregulation of certain molecules (namely NOS) in the contralateral dorsal horn. However, this mechanism no longer seems sustainable because in the monoarthritic model used here the contralateral hindpaw did not show gross inflammatory alterations [22].

The administration of either NMDA antagonist, CPP or ketamine, produced similar changes in NOS expression in the dorsal horns of monoarthritic rats; that is, a decrease in nNOS but increases in iNOS and eNOS. In this regard, it is tempting to speculate that the decrease in nNOS expression induced by the intrathecal administration of CPP or ketamine could be related to the depressant action of these drugs on the activity of pain-transmitting dorsal horn neurons, but there are no conclusive data on this matter. For example, ketamine depresses the expression of c-Fos protein (an index of neuronal activation) in various brain areas [33], but its effect on c-Fos expression in dorsal horn cells is still unexplored. In contrast, the intrathecal injection of NMDA blockers, such as CPP and ketamine, in rats exerts a rapid (a few minutes) but brief (about 1 hour) antinociceptive effect [34-38], whereas in the present study the action of these drugs on NOS expression persisted for more than 24 hours, thus indicating that changes in NOS expression resulted from genomically mediated mechanisms occurring downstream of NMDA receptor blockade, an effect that clearly outlasts the antinociceptive action of the drugs. The fact that blockade of spinal NMDA receptors produced a substantial decrease in nNOS only 6 hours after intrathecal injection of the NMDA antagonists reveals an active and fast turnover of this constitutive enzyme in dorsal horn cells. Importantly, the present results indicate that the decrease in dorsal horn nNOS after NMDA receptor blockade was accompanied by simultaneous increases in iNOS and eNOS, suggesting compensatory interactions in the expression of the different NOS isoforms.

With regard to iNOS, it has been pointed out that nNOS inhibition could activate the nuclear factor NF-κB, which may lead to the induction of iNOS [39,40] through transcriptional activation of the genes encoding iNOS [41]. Conversely, iNOS knockout mice showed increased expression of nNOS in the lumbar enlargement in comparison with wild-type mice 24 hours after challenge with carrageenan [13]. With regard to eNOS, it has been shown that endothelial NOS expression in the spinal cord of nNOS knockout mice was upregulated compared with that in wild-type mice [28]. The apparent compensation for the decrease in nNOS by rapid increases in iNOS and eNOS that we observed after administering CPP or ketamine is expected to have a functional meaning because all NOS isoforms are involved in producing the pronociceptive mediator nitric oxide, which may be relevant to prolonged treatment of chronic pain conditions, such as arthritic pain, with drugs that block the NMDA receptor.

Taken together, the present results suggest that blockade of spinal cord NMDA receptors by competitive and uncompetitive antagonists produces complex regulatory, genomically mediated, rapid (less than 6 hours) but long-lasting (more than 24 hours) changes in the expression of NOS isoforms in monoarthritic rats that may have some relevance for nitridergic neuronal/glial mechanisms involved in the pathophysiology of monoarthritis and in the pharmacological response to drugs interacting with NMDA receptor-dependent transduction pathways. In fact, it has been reported that L-NAME dose-dependently inhibits wind-up activity in the spinal cord of monoarthritic rats but not in normal controls [20], suggesting that NOS-dependent nitridergic mechanisms have a non-significant role in acute pain, whereas it may be essential in chronic pain processing.

Several studies have sought to clarify the contribution of several neural components to joint injury, rather than to determine the effect of pharmacological blockade of specific NMDA receptors in rats with adjuvant-induced arthritis. For instance, the pioneer work of Levine and colleagues [42] pointed out that no one class of nerve fiber is wholly responsible for the neurogenic component of inflammation in experimental arthritis but that large-diameter and small-diameter afferents, sympathetic efferents, and central nervous system circuits that modulate those fiber systems all influence the severity of joint injury in arthritic rats. However, no further studies establishing a relationship between lesion of specific neural components (namely dorsal rhizotomy) and changes in expression of NOS in the dorsal horn have been done. In addition, no previous pharmacological studies exploring the modifications of NOS expression in the lumbar spinal cord of arthritic animals had been performed with NMDA antagonists, even though pharmacological modulation of NOS proteins may be relevant for the successful treatment of long-lasting painful conditions, such as arthritic pain. Although the present study provides new evidence on this subject, several questions remain unanswered, such as the functional significance of decreased nNOS together with increased iNOS/eNOS after NMDA receptor blockade in spinal cord of monoarthritic rats, because no studies have examined whether nNOS loss and its possibly associated antinociceptive effect could be functionally compensated for by increases in iNOS and/or eNOS.

## Conclusion

CFA-induced monoarthritis resulted in increased expression of nNOS, eNOS and iNOS in the dorsal horns ipsilateral and contralateral to the inflamed hindpaw. Intrathecal administration of the NMDA receptor antagonists CPP and ketamine rapidly decreased nNOS expression in the spinal cord of monoarthritic rats and increased the expression of eNOS and iNOS. Results suggest that blockade of spinal cord NMDA receptors produces complex regulatory, genomically mediated, rapid but long-lasting changes in the expression of NOS isoforms in monoarthritic rats that may be relevant for nitridergic neuronal/glial mechanisms involved in the pathophysiology of monoarthritis and in the pharmacological response to drugs interacting with NMDA receptor-dependent transduction pathways.

## Abbreviations

CFA = complete Freund's adjuvant; CPP = (±)-3-(2-carboxypiperazin-4-yl)-propyl-1-phosphonic acid; eNOS = endothelial nitric oxide synthase; iNOS = inducible nitric oxide synthase; L-NAME = *N*^ω^ω-nitro-L-arginine methyl ester; NMDA = *N*-methyl-D-aspartate; NOS = nitric oxide synthase; nNOS = neuronal nitric oxide synthase.

## Competing interests

The authors declare that they have no competing interests.

## Authors' contributions

CI and MD performed most of the experiments. TP performed experiments in inducing monoarthritis. CI, AH, LC and TP conceived the study and participated in the design, in the interpretation of results, and in drafting the manuscript. All authors read and approved the final manuscript.
